# Croatian smoke-free law and smoking habits among employees of health care facilities in Koprivnica-Križevci County 

**DOI:** 10.3325/cmj.2013.54.407

**Published:** 2013-08

**Authors:** Davorka Gazdek, Senka Samardžić

**Affiliations:** 1 Department of Public Health and Social Medicine, Public Health Institute of Koprivnica-Križevci County, Koprivnica, Croatia; *szjz@kc.t-com.hr*; 2 Department of Public Health, Institute of Public Health of Osijek-Baranja County, Osijek, Croatia

Health consequences of tobacco smoke exposure are well documented and include cancer, cardiovascular disease, and respiratory disease (1). However, a significant body of evidence demonstrates that smoke-free laws are effective in protection from harmful effects of second-hand smoke and can also influence smoking behavior and smoking norms (2).

Comprehensive national smoke-free laws in Croatia were put into effect in December 1999 and since then smoking has been prohibited in all health care facilities (3). The aim of study was to investigate smoking habits trends among different professional groups of employees in health care facilities of Koprivnica-Križevci County, particularly the impact of smoking ban at the workplace compared to other tobacco control measures, such as higher taxes and anti-smoking campaign.

We explored smoking habits among the employees of health care facilities in Koprivnica-Križevci County (115 582 inhabitants). There were 1147 employees (response rate of 44%) in 1998; 1246 (response rate of 50%) in 2002; 1371 (response rate of 44%) in 2006; and 1023 (response rate of 68%) in 2011. A self-administered, anonymous questionnaire created for the purposes of this research (supplementary questionnaire(web extra material 1)) was used before and after implementation of the Act (in June 1998 – a year and a half before implementation, and February 2002, 2006, and 2011 – 2, 6, and 11 years after the implementation). The employees were divided according to age, sex, and professional groups. Health workers included physicians, dentists, pharmacists, other health-related professionals with a university diploma (psychologists, speech therapists, social workers, biochemists, sanitary engineers), and nurses. Non-health workers included administrative and technical staff (economists, lawyers, computer scientists, maintenance workers-cleaners, ancillary staff, accountants). Information on smoking status, number of cigarettes smoked, smoking initiation, intention to quit, and previous attempts to quit were collected. According to smoking status, participants were divided into three groups: current smokers, ex-smokers, and never smokers.

The questionnaire was administered to the department heads who handed them out to other employees. Respondents dropped the questionnaires in a box in department head’s office. The questionnaires were returned to researchers by post or internal mail after seven to fourteen days. There were no repeated requests to fill out surveys. The processing and analysis of data were conducted using the Microsoft Excel 2010. The data were statistically analyzed using χ^2^ test; *P* < 0.05 was considered significant.

Between 1998 and 2011, smoking rates among employees in health care facilities in Koprivnica-Križevci County decreased by 7.9% (from 34.3% to 26.4%) (Table 1). In the general Croatian population in the 1994-2005 period, smoking rates decreased by 5.2%: from 32.6% from 1994-1998 to 27.4% from 2002-2005 (4). Another study on smoking prevalence in Croatian population 18-65 years old in the 2003-2008 period found a decrease of 3.6% (from 33.2% to 29.6%) (5).

**Table 1:** Trends in smoking prevalence by professional groups and percentage of change

Characteristics	1998*	2002	Percent change 1998-2002	2006	Percent change 2002-2006	2011	Percent change 2006-2011	Percent change 1998-2011
Sex								
total	34.3	31.4	-8.5	27.2	-13.4	26.4	-2.9	-23.0
male	-	34.8	-	31.6	-9.2	24.6	-22.2	-29.3†
female	-	30.8	-	26.7	-13.3	26.8	0.4	-13.0†
Age								
<45	-	32.6	-	29.4	-9.8	28.0	-4.8	-14.1†
≥45	-	29.4	-	24.4	-17.0	25.1	2.9	-14.6†
Professional groups								
health workers	33.4	30.1	-9.9	25.8	-14.3	26.4	2.3	-21.0
non-health workers	39.2	35.5	-9.4	33.9	-4.5	26.4	-22.1	-32.7
physicians	28.3	23.5	-17.0	20.0	-14.9	19.4	-3.0	-31.4
nurses-all	35.8	33.4	-6.7	29.7	-11.1	29.4	-1.0	-17.9

*The questionnaire used in 1998 did not have any questions related to age and sex.

†Percent change 2002-2011.

In our study, the smoking prevalence in all professional groups had a downward trend, although different professional groups showed different intensity of decline. The decrease was greater among non-health (from 39.2% to 26.4%) than health workers (from 33.4% to 26.4%), and among physicians (from 28.3% to 19.4%) than nurses (from 35.8% to 29.4%).

Smoking prevalence among employees in health care facilities in Koprivnica-Križevci County had a downward trend, as was the case in the general Croatian population. However, smoking prevalence among employees in health care facilities in Koprivnica-Križevci County decreased considerably more and was lower than in the general Croatian population, which could be explained by the impact of the smoking ban in health care facilities.

Our study implies that smoking ban at the workplace had a positive impact on smoking reduction among employees in health care facilities. The average number of cigarettes per person decreased from 15 to 12 cigarettes per day. The percent of people who smoked 10 or fewer cigarettes per day increased from 33.7% to 57.4% and the percent of people who smoked more than one pack of cigarettes per day decreased from 15.9% to 6.8%, and this decrease occurred mostly in the first two years after the implementation of the Act. This is in agreement with previous studies, which showed that comprehensive clean indoor laws lowered per capita cigarette consumption by 5%-20% (6,7). Moreover, another study showed that implementation of totally smoke-free workplace was associated with 3.1 (2.4 to 3.8) fewer cigarettes smoked per day per continuing smoker (8). Other studies also found that totally smoke-free workplaces reduced the prevalence and increased quitting (2,9), as well as reduced cigarette consumption by 2-4 cigarettes per day per person (2,10).

The smoking ban at the workplace also affected smoking behavior relative to the time passed since its implementation. In the first two years after the implementation, the smoking prevalence decreased by 2.9% (8.5% reduction), and after 2 to 6 years it decreased by 4.2% (13.4% reduction). Another study found that totally smoke-free workplaces were associated with a decrease in the prevalence of tobacco consumption by 3.8% within two years of implementation (8). It was also found that worksite regulations reduced smoking prevalence by 7 to 20% one year or more after implementation (6), with a strong long-term effect (7).

In our study, smoking ban at the workplace had different effects in different professionals groups. The decrease was greatest between 2 and 6 years after implementation of the Act among health workers, and between 6 and 11 years among non-health workers. The greatest decrease in smoking prevalence among physicians was recorded in the first two years after the Act came into effect and the greatest decrease among nurses was recorded after 2 to 6 years. This is in accordance with a study that showed that after implementation of the ban physicians were more likely to quit smoking than nurses (11). Another study showed that women with a low level of education were particularly responsive to media messages and to the increase in the price of cigarettes, especially compared with highly educated women (12).

In a study by Levy and Friend (8), most of the 15 individual studies on smoking prevalence after implementation of total or partial smoke ban in US hospitals showed a positive impact on smoking prevalence reduction. A recent systematic review (9) found that higher tobacco prices, smoking bans in public places, and anti-tobacco mass media campaigns had a strong independent effect on smoking prevalence, while health warning labels and bans on advertising and sponsorship had only a limited effect.

Although we cannot make any conclusion on the causal relationship between smoking ban at the workplace and smoking behaviors in our participants, there are strong indications that a smoking ban is a more effective measure to reduce smoking than other tobacco control interventions (7,9). Several studies in Croatia were primarily focused on using tax-based policies to reduce tobacco consumption. They showed that while Croatia had made good progress in adopting tax-based policies to control tobacco consumption, black market sales of cigarettes were still large, the penalties for non-compliance with the law minor, cigarette prices and taxes low (13), and tax policy impact on consumption of tobacco products small and limited (14). Another study concludes that in some aspects of tobacco use and regulation Croatia fares better than other European countries because more Croatian smokers and ex-smokers have been exposed to anti-smoking campaigns than smokers in other European countries. However, in other aspects it is somewhat lagging behind because the effectiveness of such exposure is modest in terms of the percentage of smokers who wanted to quit smoking and the relatively low share of population that is protected from second-hand smoke (15). However, the anti-smoking campaign in Croatia was particularly strong in the period 2002-2003, when Croatia established the national center for the prevention of smoking and national telephone smoking helpline, organized Croatian “Smoke Out Day,” the Croatian National Television broadcasted anti-smoking spots (16-18), and the WHO anti-smoking campaign “Quit and Win” was launched including famous Croatian persons (19). The celebration of World No Tobacco Day-2003 gathered more than 10 000 people (20). Although World No Tobacco Day and “Quit and Win” campaign have been regularly marked in Croatia, there have been no national mass media campaigns since 2003 (21).

The smoke-free legislation is expected to produce significant reductions in environmental tobacco smoke exposure. Moreover, there are likely to be additional, important public health benefits by facilitating smoking cessation, reducing smoking prevalence, changing cultural attitudes toward smoking, and reducing smoking-related morbidity and mortality. Much progress has been made in reducing tobacco use in Croatia in the last decade by introducing comprehensive smoke-free legislation. Smoking ban at the workplace had a positive and long lasting impact on the smoking prevalence among employees in health care facilities in Koprivnica-Križevci County. To continue the fight against smoking it is necessary to increase taxes and prices of tobacco products, implement control measures, and introduce comprehensive cessation programs.

## Additional Material

Supplementary questionnaire
