# Supplementary material for: Croatian smoke-free law and smoking habits among employees of health care facilities in Koprivnica-Križevci County
Source: Croat Med J. 2013 Aug;54(4):407–10. doi: 10.3325/cmj.2013.54.407 (PMC3760667; doi:10.3325/cmj.2013.54.407)
Supplement: Supplementary questionnaire [file CroatMedJ_54_s004.pdf]

QUESTIONNAIRE

Smoking habits among employees in health care facilities in Koprivničo-Križevačka county

Do you smoke? (Please circle)

YES

A) Occasionally

B) Daily

How many cigarettes do you smoke per day?__________

When did you start smoking? (age)___________

Have you ever tried to quit smoking? YES NO

NO

A) I have never smoked

B) I used to smoke

When did you quit smoke? (year)

____

Gender: A) male B) female

Age:____________

Profession:______________________

Education:
A) University degree
B) College
C) Secondary
D) Primary
E) Other___________

Health care facilities:
A) Hospital
B) Health care centre
C) Department of public health
D) Private practice
E) Pharmacy
F) Other_____________________

Thank you for your answers!
